# Staff resilience and innovation essential to New York City diabetes prevention programs going virtual during COVID-19 pandemic lockdowns

**DOI:** 10.1186/s12913-023-10129-y

**Published:** 2023-10-25

**Authors:** Eleanor J. Smith, Leora J. Apfelbaum, Ming-Chin Yeh, Margrethe F Horlyck-Romanovsky

**Affiliations:** 1 Teachers College, Columbia University, New York, NY USA; 2 Nutrition Program, Hunter College, City University of New York, New York, NY USA; 3 Department of Health and Nutrition Sciences, Brooklyn College, City University of New York, Brooklyn, USA

**Keywords:** National Diabetes Prevention Program, Type 2 Diabetes Mellitus, COVID-19 pandemic, Program operation, Diabetes prevention, Virtual programming

## Abstract

**Background:**

COVID-19 lockdowns in March 2020 forced National Diabetes Prevention Programs (DPPs) to pause, cancel or reformulate. This qualitative study sought to (a) document if/how New York City(NYC) DPPs adapted and served participants during lockdowns, and (b) identify successes and challenges to operating programs during the lockdowns and restrictions on social gathering.

**Methods:**

Researchers contacted 47 CDC-registered DPPs in NYC. Eleven DPP directors, lifestyle coaches, and coordinators involved in program implementation completed 1-hour semi-structured virtual interviews and received a $50 gift card. Interviews were recorded, transcribed, and analyzed using Grounded Theory (Dedoose, Version 9).

**Results:**

Interviewees represented 7 organization types: public hospitals, weight loss programs, healthcare centers, community-based organizations, health insurance companies, faith-based DPPs, and federally qualified health centers. DPPs served participants in 4 of 5 NYC boroughs. Six organizations provided DPP services during lockdowns by going virtual. Successes and challenges related to staffing, resource allocation, virtual data tracking, and participant engagement. Most programs were successful due to resilient, dedicated, and extraordinarily innovative staff.

**Conclusion:**

The pandemic highlighted opportunities for successful virtual DPPs in urban settings, and the need for more robust funding, staff support, and technical assistance for sustainability and scalability of the DPP.

**Supplementary Information:**

The online version contains supplementary material available at 10.1186/s12913-023-10129-y.

## Introduction

In New York City, 36% of adults have prediabetes, and every year an estimated 66,000 New Yorkers are diagnosed with diabetes [1, 2]. However, diabetes disproportionately affects minority populations, and rates are twice as high among Black (14%) and Hispanic (12%) New Yorkers than Whites (6%) [3–6]. In 2020–2022, the COVID-19 pandemic further highlighted the health inequities and disproportionate diabetes burden among New Yorkers of color. COVID-19 mortality rates were twice as high (248/100,000) for Black New Yorkers compared to Whites (123/100,000) [6, 7]. Blacks and Hispanics were more than twice as likely to be hospitalized than Whites despite having similar underlying conditions [8, 9]. Underlying conditions such as diabetes (58%) and heart disease (73%) contributed to 85% of all COVID-19 deaths [6, 7].

The COVID-19 pandemic also impacted diet and physical activity. Research findings on the pandemic’s effect on dietary habits are mixed. Some international studies reported increased fresh produce consumption and home cooking, whereas others reported decreased consumption of fruits and vegetables and increased snacking and meal frequency [10]. Overall physical activity (measured by IPAQ score) decreased and more significantly in low-income communities [11, 12]. Therefore, the need for innovative and effective healthy lifestyle programs may be even greater now than before the COVID-19 pandemic.

The Centers for Disease Control and Prevention’s (CDC) DPP is a nationally recognized lifestyle intervention program recommended for people who are at risk of diabetes to support them in preventing the disease. Between 2012 and 2021, a total of 2,201 host organizations were recognized by the CDC to provide the DPP, and more than 569,603 people had participated [13]. Recognized host organizations may be approved to deliver sessions either “In Person”, “Online”, through “Distance Learning”, or a “Combination”. Host organizations can have the following recognition status: “Pending”, Preliminary or Full. Only Preliminary or Full recognition status allow organizations to bill Medicare and host Online, Distance or Combination sessions. Certified DPP lifestyle coaches enroll eligible participants and deliver sessions. Participants and lifestyle coaches meet for 16 weekly sessions and then monthly during a 6 month follow-up period. The DPP has been shown to reduce the risk of developing diabetes by 58% [14]. Indicators of success of the DPP are a reduction in hemoglobin A1c, a minimum of 5% weight loss, and an increase in physical activity above 150 min per week. During the Covid-19 pandemic, the CDC granted permission for all programs to deliver online or distance learning models regardless of recognition status.

On March 1, 2020, the first COVID-19 case was confirmed in New York City. By March 16, 2020, New York City public schools, colleges and universities converted all education to remote sessions, many workplaces converted non-essential workers to work from home, and limitations on social gatherings were implemented. The pandemic lockdowns also forced community-based National Diabetes Prevention Programs (DPP) to pause, cancel or reformulate their programming [15]. On March 18, 2020, the CDC announced that regardless of approval status, all DPP sites could convert their sessions from in-person to online without affecting their recognition status and ability to get reimbursed for their services. Across the US, various degrees of pandemic lockdowns soon followed. Studies examining the effect of the pandemic on DPPs include a national qualitative case study by the CDC [16], a qualitative study of Los Angeles Couty DPPs [17], and a survey of community pharmacies [18]. These studies showed that DPPs across the United States shared the following challenges a) COVID-19 related staff shortages; b) organizational and individual lack of access to and/or knowledge of technology; c) data collection hampered by lack of access to safe online platforms and/or poor internet access; d) extraordinary efforts were needed to collect data from and to connect with participants, including loaning digital devices; e) participants and lifestyle coaches lamented the absence of personal interaction both for recruitment and retention purposes; and f) reporting/reimbursement were onerous, reimbursement rates are inadequate to sustain the program [19, 20], and therefore a majority of programs did not report to the CDC nor did they bill Medicare/Medicaid or private insurance [18]. Furthermore, they found that enrollment was affected initially, but as telehealth options became desirable and more convenient enrollment improved. Findings suggest that the transition to telehealth was challenging in similar ways for most United States DPP providers [17]. Because of its lower cost, ability to reach long distances virtually, and potential efficiency when employed as part of a hybrid approach, all three studies concluded that online or hybrid delivery modalities remain viable, offering benefits beyond the traditional program models.

However, due to the early and disproportionate impact of the COVID-19 pandemic in New York City, it is unclear how New York City DPP sites adjusted their programs during the 2020 and 2021 period, and how these changes affected program participation, engagement, and outcomes. Therefore, using a qualitative approach, this study sought to a) document if/how New York City DPPs adapted and served participants during pandemic lockdowns, and b) identify successes and challenges to operating a DPP during the public health emergency and pandemic restrictions on social gathering.

## Materials and methods

**Fig. 1:**
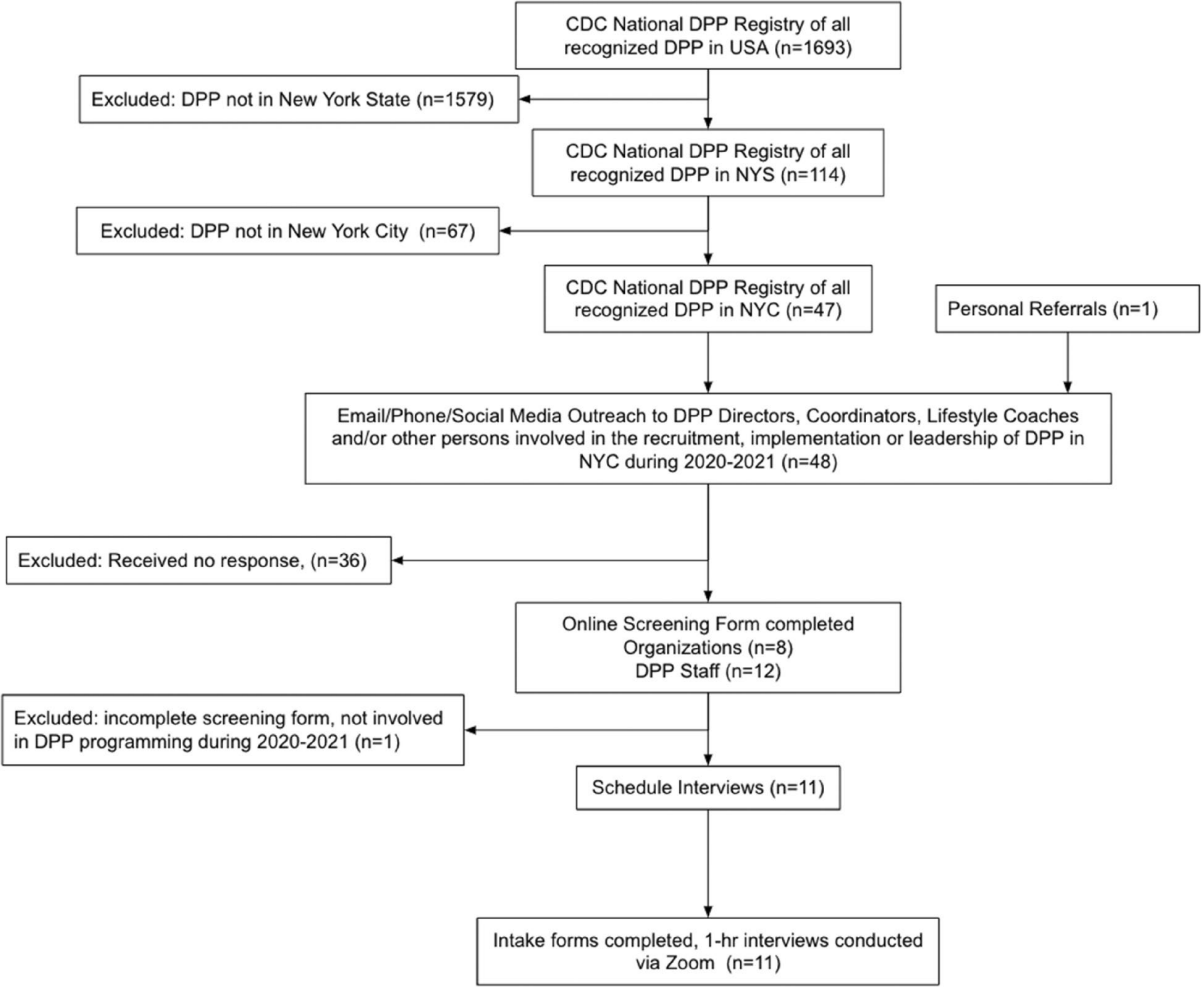
Flow diagram of recruitment of New York City Diabetes Prevention Program staff who were active during the COVID-19 pandemic

Using the National Registry of Recognized Diabetes Prevention Programs of 1,693 organizations recognized by the CDC to deliver the DPP in the United States as of August 18, 2020, we selected all programs in New York state (Fig. 1). Of the 114 DPPs in New York State in the CDC DPP Registry as of August 18, 2020, 47 had an address in New York City. Between March and October 2021, our team reached out to all New York City DPPs. Recruitment methods included phone calls, emails, Twitter, LinkedIn, and personal referrals and each site was contacted on multiple occasions. We aimed to recruit program directors, managers, and lifestyle coaches directly involved in the planning, implementation, and delivery of the DPP during the pandemic lockdowns in 2020 and beyond. A total of 12 potential participants representing 8 DPPs completed a screening questionnaire online or over the phone. One site was ineligible because it was not involved in DPP activities during the study period. A total of 11 participants representing 7 different DPP host organizations (a community based organization, a citywide multi service provider, a public hospital, a regional health insurance company, an independent contractor serving houses of worship, a national lifestyle company, and a network of federally qualified health centers) proceeded to an interview. DPP host organizations which did not respond or participate in the study included 12 public/private hospitals, 6 community based organizations, 7 private practice (doctors, registered dietitians and health educators), 4 national lifestyle companies/apps, 3 health clinics, 2 city agencies, 2 religious institutions, 2 worksite wellness providers, 1 diabetes management app, and 1 fitness center. In terms of borough distribution of the non-participating sites, 4 were in the Bronx, 13 were in Brooklyn, 15 were in Manhattan, 7 were in Queens, and 1 was in Staten Island.

### Semi-structured interviews

Oral informed consent was obtained at the beginning of each interview. Interviewees were assigned a unique 6-digit identifier to maintain participant confidentiality.

A quantitative online intake survey developed by the study team gathered basic information about the DPP such as recruitment methods, session delivery method before and during the pandemic, enrollment, and primary target population(s). To ensure validity, survey questions used standard language and categories extracted from CDC Diabetes Prevention Recognition Program Standards and Operating Procedures [21], e.g. delivery method (In-person, Online, Distance Learning or Combination), organization type, and nativity of program participants.

The qualitative component consisted of semi-structured one-hour interviews which were conducted via Zoom. As the DPP staff roles in some cases overlap and some interviewees were titled directors or coordinators while also serving as lifestyle coaches, we used the same general questions for everyone. Interview questions focused on understanding pre-pandemic operation of the DPP, and any interruption, closure, or reconfiguration due to COVID-19 lockdowns. Interviewees were asked about indicators of success within their programs, e.g., HbA1c reductions and weight loss, methods of remote data collection, challenges, and opportunities during the COVID-19 lockdowns. Interviews were conducted by MHR, ES, or LA, recorded, and transcribed verbatim using Zoom live transcription. Interviewees received a $50 electronic gift card immediately after the interview.

### Analysis

Interview transcripts were deidentified, coded, and analyzed using Dedoose 9.0.46. Using grounded theory methodology [22–24], a dynamic and iterative process in which data collection and analysis alternate, the data collected through in-depth interviews informed our analysis which in turn informed concepts or emerging themes to pursue in subsequent interviews. Three research team members participated in initial coding of transcripts and generating a codebook. We used in-vivo codes, actual words of participants, to label concepts. All transcripts were coded by two team members, and any discrepancy was resolved by a third team member. The team compared coding choices until consensus was reached. Throughout the study, the research team discussed emerging themes and wrote memos to move the analysis forward. Saturation was reached when no new data emerged from additional interviews [23, 24]. After the analysis, we organized the themes in domains expected to be informative for future DPP conversion or implementation of online program interventions. Themes were organized as opportunities and challenges grouped by the following domains: Going Virtual, Staffing, Training, Recruitment and Retention, Educational Materials and Sessions, Communication, Data Collection and Tracking of Body Weight, and Funding.

The study was conducted in accordance with the Declaration of Helsinki and deemed exempt by the Human Research Protection Program of the City University of New York.

## Results

### Program characteristics

In total, 12 representatives from 8 different DPP provider organizations completed the online screening form, and 11 staff from 7 organizations completed an interview (Table 1). Sites represented DPPs in four of the five boroughs of New York City: Manhattan, Brooklyn, Queens, and the Bronx. Provider categories included a community based organization, a citywide multi service provider, a public hospital, a regional health insurance company, an independent contractor serving houses of worship, a national lifestyle company, and a network of federally qualified health centers. Two organizations saw an increase in participants, one saw no change in enrollment, one started the program during the pandemic, one organization’s DPP remained suspended, and two organizations experienced a decrease in enrollment during the early COVID-19 pandemic. Recruitment methods remained the same before and during the pandemic. All DPPs in this study served primarily low-income minority populations. The programs’ main racial and ethnic groups differed by program, and some programs offered their courses in languages besides English. Racial and ethnic groups served by the DPPs interviewed include Bengali, Black Americans, Chinese, Dominican, Ecuadorian, Haitian, Korean, Mexican, Puerto Rican, Salvadorian, Spanish-Caribbean, and White Americans (Table 1). The share of foreign-born DPP participants ranged from 20 to 100%.

**Table 1:** Diabetes Prevention Program Characteristics, Pre-Covid and During-COVID: Program Details, and Populations Served

Site	Host Organization Type	Suspension Duration	Pre-COVID Delivery Mode	During COVID Delivery Mode	Pre-COVID Cohort Size	During COVID Cohort Size	Recruitment Methods	Racial/ Ethnic/Cultural Groups	Foreign-Born Participants
**Programs that were suspended during the pandemic**									
1	Community-Based Organization	2 months	In-Person	Distance Learning	40	Increased	-Advertisements -CBO referrals -In-person outreach/flyers -Social media/Word of mouth	-Korean	100%
2	Citywide Service Provider	3 months	In-Person	Distance Learning	20	Decreased	-Advertisements -CBO referrals -Hospital referrals -In-person outreach/flyers	-Ecuadorian -Mexican -Salvadorian	90%
3	Hospital, Out-patient	2 months	In-Person	Distance Learning	15	Decreased	-Advertisements -CBO referrals -Hospital referrals -In-person outreach/flyers	-Black -Dominican -Puerto Rican -White American	40%
4	Health Insurance Company	2 years	In-Person	Still suspended (preparing distance learning)	25	N/A	-Advertisements -CBO referrals -In-person outreach/flyers -Word of mouth	-Black -Chinese -Dominican -Mexican -Puerto Rican -White	50%
**Programs that were NOT suspended during the pandemic**									
5	Faith-Based	N/A	In-Person / Distance Learning	Distance Learning	12	Increased	-Advertisements -In-person outreach/flyers -Physician referral -Social media/Word of mouth	-Black -Dominican -Ecuadorian -Puerto Rican	25%
6	For-Profit Lifestyle Company	N/A	In-Person	Combination	12,000	Stayed the same	-Online recruitment platform	-Unknown	Unknown
**Programs that started during the pandemic**									
7	Federally Qualified Health Center	Started during the pandemic	N/A	Distance Learning	N/A	N/A	-In-person outreach/flyers -Physician referral -Phone calls to newly Dx -Ads on website	-Black -Bengali -Haitian -Spanish-Caribbean -White	20–50%

In-Person: Delivered 100% in-person for all participants by trained Lifestyle Coaches; Online: Delivered 100% online for all participants who log into course sessions via a computer, tablet, or smartphone; Distance Learning: Delivered 100% by trained Lifestyle Coaches via remote/asynchronous classroom; The organization must be able to track the participants’ progress through online course sessions. CDC recommends requiring user IDs and passwords for course access. Live Lifestyle Coach interaction is required and should be offered to each participant no less than once per week during the first six months and once per month during the second six months. Emails and text messages can count toward the requirement for live coach interaction as long as there is bi-directional communication (i.e., organizations do not simply send out an announcement via text or e-mail and count that as live coach interaction; the participant must have the ability to respond to and get support from the live coach)

#### Interviewee characteristics

We interviewed 3 directors, 3 coordinators and 5 lifestyle coaches. All program providers were CDC certified lifestyle coaches. Of the 11 interviewees, 7 had relevant credentials: 5 were Registered Dietitian Nutritionists (RDN), 1 RDN was also a Certified Diabetes Care and Education Specialist (CDCES); 1 lifestyle coach had a Master of Public Health (MPH); and 1 lifestyle coach was a Certified Health Education Specialist (CHES). DPP staff had between 4 months and 8 years of experience implementing the CDC’s DPP.

### Qualitative results

Themes and sample quotes are organized as opportunities and challenges grouped by the following domains: Going Virtual, Staffing, Training, Recruitment and Retention, Educational Materials and Sessions, Communication, Data Collection and Tracking of Body Weight, and Funding.

#### Going virtual, educational sessions, and materials

##### Challenges

Theme: Organizations struggled to deliver the workshops online, connect with participants and identify safe virtual platforms.

At the beginning of the pandemic lockdowns, 4 of the programs in this study were asked to suspend the program, while 2 immediately transitioned to working remotely. Only the hospital-associated programs appeared to be affected by staff reassignment to COVID-19 related care. Starting in April 2020, the CDC allowed all sites to convert to virtual sessions to facilitate the continuation of the DPP. As New York City was adjusting to the pandemic reality, staff did not have access to the equipment necessary to deliver DPP and instead depended on personal computers and cellphones.

As DPP programs considered converting to virtual sessions, some were able to adopt Zoom, Microsoft teams, or WebEx. However, HIPAA regulations prevented healthcare sites from using common virtual platforms, which delayed the conversion for DPP sessions to remote operation.

Programs that implemented virtual sessions, staff reported struggling to engage participants. One interviewee mentioned the difficulties in getting participants to engage in a group session, stating:

*Lifestyle Coach B: In in-person class, they are very active [they talk about themselves and how they are really concerned about their diabetes] or their successes […] but in virtual class they just listen. It’s a little hard because they still have difficulties operating Zoom and the mute/unmute function.*

An important part of in-person sessions is that participants share experiences and stories which validate and serve as motivation for others. Several lifestyle coaches noted that this essential part of the DPP was lacking in the virtual setting as participants are uncomfortable or unable to unmute during the virtual setting. DPPs sometimes provide food and giveaways during in-person sessions to incentivize participation and mark milestone achievements. Interviewees noted that for obvious logistical reasons this was not possible in a virtual setting and felt that this may have contributed to lower participant engagement and higher attrition rates during the pandemic. One coordinator mentioned that virtual activities decreased engagement and excitement in the program, saying:

*Lifestyle Coach A: It’s the little things that [would keep people interested] and those we had to omit because of COVID. Given that the program would have people more engaged, [we would] take a trip to the supermarket and or farmers market [or] bring healthy snacks in for them.*

Although participant handouts are available in several foreign languages, the lifestyle coach manual is only available in English and Spanish. Therefore, sites that served specific populations spent time translating material for the PowerPoint slides used during the virtual sessions.

*Coordinator B: All of our materials are in English, some [are in] Spanish. That is a barrier not having more specific kinds of languages we don’t necessarily have materials available in Mandarin or Vietnamese.*

##### Opportunities

Theme: Virtual DPP sessions showed promise for the urban setting where commute time, competing schedules, and limited income often precluded attending in-person sessions during non-pandemic times.

Once the DPPs were able to resume virtual sessions, participants were able to attend sessions that met their preferred language or cultural preferences without having to travel. Thus, virtual DPP sessions showed promise for the urban setting where commute time, competing schedules, and limited income often precluded attending in-person sessions during non-pandemic times. In fact, participant enthusiasm and enrollment increased for 2 programs because of the ability to attend sessions from home or work with no need to travel. One interviewee noted,

*Lifestyle Coach B: We have Brooklyn clients; we have Staten Island clients [and they access the DPP] remotely because it is a virtual classroom.*

The DPP program coordinator in a public hospital noted that virtual programming specifically benefited low-income individuals, stating,

*Lifestyle Coach D: A lot of these participants are somewhat tight with money and even commuting to one of the centers could be a bit of a challenge… The flexibility [of online classes allows them] to take the class during their lunch hour or while preparing meals at home.*

One host organization even started a new DPP. Because the virtual option was available to new host organizations during the CDC’s public health emergency exceptions the organization saw it as a rare opportunity to enroll hard-to-reach low-income populations at disproportionate risk of diabetes. The program director of this organization explained:

*Director C: We came into the pandemic [and] we found out that there was the option to do [the DPP] virtually. We had started doing some virtual classes already for our patients, [like] virtual yoga for free, virtual cooking classes, and we had success with attendance. [...] We were surprised that we got a good cohort, and not only that, but they were consistently coming.*

In addition, three interviewees observed that future generations at risk of diabetes may be more likely to attend a virtual program rather than in person sessions.

*Coordinator B: Especially for younger populations like it's, In full disclosure, I will say I'm a millennial in my mid-30s. I can’t imagine going to a conference room at a hospital or Y facility for an hour and a half class for a year.*

#### Staffing and training

Theme: The amount of staff time necessary to run a DPP program whether in person or virtually is hard to estimate, but likely far more than may be expected.

##### Challenges

Theme: Outstanding staff efforts and participant enthusiasm made it possible for most DPPs to quickly convert to virtual sessions.

During the pandemic, health care staff who were usually assigned to DPP were understandably reassigned to essential COVID-care. Two interviewees from a hospital program stated:

*Lifestyle Coach E: I am a dietitian and I have a full clinic to cover. The other lifestyle coach is a nurse assistant [...] he was at a COVID floor so he couldn’t do any of this with me.*

*Coordinator A: Not being able to control [my] staff’s schedules, like you can’t run a program for an hour and think that’s the only time you need for the program. You need prep, you need follow up. You need to have time and [...] and they’re just not given admin time, it’s awful.*

Although many DPP staff had never used virtual platforms, let alone used them for leading health education programs, those who were able to teach themselves, each other, and DPP participants how to install and operate various platforms. Staff created educational slides and translated messages to specific languages to best accommodate their audiences. Throughout the interviews it was clear that DPPs only succeeded in continuing or resuming the DPP because the staff were dedicated to the program and its participants and that participants were also willing to make it work. One program director stated,

*Lifestyle Coach B: It was a great teamwork and then each lifestyle coach was passionate even though it's very hard…New York city [was] hardest hit by the Covid-19, but even though we really a little bit scared to go out to meet someone in person [...] we were brave and courageous.*

When asked about how much of their time was dedicated to recruitment, preparation of sessions, reminder phone calls, and following up with participants who were unable to attend, interviewees said, “a lot” and “an extraordinary amount of time”. Several lifestyle coaches discussed dividing up the cohort among them to be able to follow up with everyone. When asked about an approximate participant/lifestyle coach ratio, many said that they could each support 3–5 participants. None of the programs had more than a couple of lifestyle coaches and were therefore not adequately staffed to accommodate cohorts of 15–20 participants.

One interviewee mentioned that administrative support could potentially make a significant difference in the workload and participant success across the program, saying:

*Coordinator A: Say we had like 15 people. Someone needs to call 15 people and say, have you used Webex or whatever platform, are you comfortable with it, because it takes so much time. [...] I think clerical type support would be the best.*

Extensive staff training was also needed to ensure that non-essential workers could work from home, but also to resume the DPP and other health promotion services. However, participants also needed digital mentoring, which had to be done remotely.

*Lifestyle Coach B: In late March, we had a virtual coach meeting, so we decided to take responsibility [on]how to work with the clients. But even though the coach members are around 50 and 60 so this is why they could understand how difficult, even though they didn't know what Zoom is.*

##### Opportunities

Theme: Health Departments were able to provide technical support.

Five sites also reported receiving technical support and advice from the New York City Department of Health, the New York State Department of Health, as well as the CDC. One interviewee expressed their gratitude, stating,

*Lifestyle Coach A: The Department of Health and Mental Hygiene [have] helped us tremendously. They got us trained and I actually have [monthly meetings with] one of their workers. She helped me with the process of getting certified for Medicaid. Any time I have any issues, whenever I have to submit data to the CDC, they’re great so, they got us trained and they’re still here, helping us.*

#### Recruitment, retention, and communication

Theme: Challenges in recruitment and retention were direct effects of the lack of social and professional interactions with physicians and health educators in public spaces and events.

##### Challenges

During the pandemic lockdown in New York City, all medical providers and facilities were redirected towards caring for COVID-19 patients, testing, contact tracing and eventually vaccinations. Non-essential facilities were either closed or repurposed. This meant that regular checkups and maintenance care were suspended. In addition, patients were fearful of visiting medical facilities for fear of infection and therefore postponed regular visits. Fewer medical visits meant that fewer patients were identified as at risk of diabetes and therefore fewer were referred to the DPPs. One lifestyle coach noted how this affected their program:

*Lifestyle Coach A: [People] couldn't get an appointment with their doctor, so they didn't remember when their last A1C was. That was a little more challenging and that's why we didn't get as many participants because we didn’t have a lot of referrals.*

In terms of DPP participation and attrition prior to the pandemic, many DPP participants may visit the facility at other times. Thus, they may have in-person chance encounters with the DPP lifestyle coaches or be reminded at their doctor’s visit to attend the next DPP session. However, during the pandemic, the lack of in-person interactions placed greater responsibility on individual participants to stay connected with the DPP, and its leadership and fellow participants which eventually led to higher rates of attrition. In addition, although recruitment appeared to be higher during the COVID-19 lockdowns, as some participants were required to return to work, attrition was higher than usual. Participants who dropped out were more likely to be younger, required to return to work or other obligations, and therefore unable to attend classes. As the pandemic lockdowns were eased, many people no longer had the flexibility of working from home, added commute time and childcare logistics resumed, and other obligations competed for time. One interviewee discussed the challenges with retention, saying:

*Lifestyle Coach C: We started with about 15 patients, it’s down to 8 now [...] I think that they’re really enjoying it, they’re coming to most of the sessions. For our clientele, there’s a bunch of different things going on in their lives, so it’s really hard for them to commit long term.*

Recruitment and retention placed a burden on DPP staff as they had to make more phone calls to enroll and retain participants. In addition, some made home visits to teach participants to use their cellphones for data collection and attendance at virtual sessions. Many also resorted to mailing materials and equipment as one program coordinator stated:

*Lifestyle Coach E: We started with phone calls. I even mailed them the curriculum, and I even mailed them the health bucks because farmers markets [were] still on.*

Sites consistently noted that phone calls were the most effective form of communication with DPP participants. Email, text messages, and messaging apps were found to be ineffective despite their convenience because not all participants used these forms of technology regularly. In addition, emailing or mailing materials to participants was costly and labor intensive. One interviewee noted:

*Coordinator A: I want to make sure I’m signing you up with your email address, and I want to know, “do you use your email?”. That’s just a step in the right direction towards if we needed to go virtual again, like [if I know your email] that tells me that you probably can do a Webex or other means of communication. The certified lifestyle coach, she was putting stuff in the mail all the time and it’s so laborious compared to just sending an email with attachments.*

In some cases, staff even taught individual make-up sessions over the phone because email and video platforms were not feasible.

##### Opportunities

Theme: The virtual setting initially increased enrollment in the DPPs because there was no need for travel, people were available, sessions could be flexible, and people were looking for social interaction.

The virtual setting initially increased enrollment in DPP because there was no need for travel, many participants were working from home, and session times could be more flexible. In addition, one host organization started a DPP because the virtual option became available to all providers and found that distance learning was more realistic for their clients.

*Director C: We were surprised that we got a good cohort and not only that, but they consistently are coming, and so it did show a huge difference compared to anything that we've ever tried to do in person at the clinics - so far it has been a … fortunate event.*

Factors that positively impacted enrollment and retention included support from referring physicians and strong social support networks that developed among DPP participants in a time of few social interactions. In addition, participant motivation increases with the support of provider teams including dietitians, doctors, lifestyle coaches and peer health educators.

*Coordinator C: [When a physician says]“Oh, I heard you’re in the DPP and you’re doing so well.” I feel like that has really helped with retention when the doctor is involved. […] Sometimes, too, how the class gels [is important]. Some people in classes become friends and they’re like “You weren’t here last week, what happened to you?”*

Communicating with participants over the phone outside of the group sessions was used to improve engagement. One lifestyle coach said:

*Lifestyle Coach D: Participation is a huge factor [to succeed] in the program. Sometimes [I] get one or two that are super shy, so I make sure to give them my direct line. [I say] Please give me a call and I will work with you every step of the way [...] I feel very passionate about this program, and I give it my all.*

Increasing digital literacy and connectedness was seen as an opportunity to continue virtual sessions for certain audiences or allowing DPP host organizations to offer a choice of in-person or virtual sessions. For example, a social media app was used to connect and communicate with participants for one program. The director stated:

*Director A: We are on a social media [app], you know, like all [members in our community] have [this app], Everybody is on [this app]. So, you know, like in DPP you know we have like a group of chatting room, so they were all in the chat room already, so we just have to let them know we got to start the class. Yeah, everybody was so excited, and they try to make a healthy plate and take a picture and show it. “Oh, I made this” and the coaches [would] make a comment “wow that looks good!”, “that’s beautiful!”*

Most providers recommended that in the future, regardless of their CDC approval status, DPP host organizations should be allowed to provide both in-person and virtual DPPs to accommodate participants with different preferences, time constraints, and access or no or low access/affinity with technology.

#### Data collection and tracking of body weight

##### Challenges

Theme: Tracking participant weight, physical activity and HbA1c data was very difficult.

The DPP requires host organizations to track participant attendance, body weight, and physical activity. During in-person sessions body weight is collected on site and physical activity is reported by participants. In response to the pandemic, almost all the host organizations purchased and shipped body weight scales to participants who did not have a scale at home. Several sites also provided fitness trackers. It was unclear to the interviewees, or they were unable to share what sources of funding were used to purchase these items.

*Director B: That has been, ironically, one of the most challenging parts of this. Getting people used to sending in your weights and activities minutes. It’s a nightmare, and I’ve even invested in the apps where [I] personally put all the information in right after the session.*

*Coordinator B: The challenge is making sure we have all that data [...]. Self-reporting weight [is] our proxy for attendance. The caveat to that was obviously folks who don’t have a scale at home.*

HbA1c test results may also be collected to track DPP participant success. In hospital and health clinic-based programs HbA1c was often extracted from electronic medical records. For community based programs, participants brought test results from their doctors. However, given that participants had fewer in-person medical appointments, HbA1c results were less likely to be available, and documentation of glycemic control was limited.

##### Opportunities

Theme: Self-reported data is not always accepted, so collecting data remotely meant that staff had to innovate.

Having to collect data remotely meant that DPP staff had to innovate and employ social media and mobile apps or online platforms to streamline data collection. Thus, the pandemic forced DPPs to transition to virtual data collection rather than paper records. Platforms for data collection included Practice Better, Wellocity, Bluetooth enabled devices, and various electronic health record systems. As one lifestyle coached noted:

*Coordinator B: There are several health insurance companies that will not accept self-reported weight, but they would accept Bluetooth scales sync to an app.*

DPP sites affiliated with medical facilities encouraged regular HbA1c tests and could obtain results through electronic medical records. As improvements in HbA1c may occur in the absence of significant weight loss such testing is crucial in demonstrating DPP impact. One interviewee highlighted the benefit of coordinating free HbA1c testing from an affiliated clinic, saying:

*Coordinator C: [affiliated clinics] would help do free A1C tests, which was really helpful especially [since] we did them week 1 and week 16. At the very end, so people can kind of see the progress, and it was helpful with the doctors.*

#### Funding and sustainability

##### Challenges

Theme: Inadequate funding, labor-intensive documentation and reimbursement requirements means that most provide the DPP for free.

Funding for the operation of DPP differed by host organization type. Community based organizations noted that they had received seed funding from the city and/or state health departments, and others noted small foundation grants. However, most organizations appeared to be operating the DPP as a free service to their clients. In fact, when asked about billing Medicare, Medicaid, and health insurance companies, almost all interviewees noted that the reimbursement process is time consuming, many participants are not technically eligible, reimbursement amounts are quite small, and therefore the process of filing for reimbursement is often not considered cost effective. In addition, the administrative burden associated with tracking and documenting participant weight loss and HbA1c progress in addition to program attendance threatens the sustainability and expansion of the program.

DPP recognition status (Preliminary, Full, etc.) depends on the amount of time a specific organization has been delivering the program, its success rates, and whether 5% weight loss is achieved by participants. To bill Medicare/Medicaid, DPP programs must have full recognition. Some sites noted that recognition requirements had become stricter. As of May 1, 2021, a 0.2 percentage point reduction in HbA1c was added as a measure of success [21]. Interviewees in medical care facilities appreciated demonstrating glycemic improvement since participants may have an easier time achieving lower HbA1c than a 5% weight loss. However, community based organizations were unable to request and obtain HbA1c results and therefore unable to document success and therefore may have difficulties achieving full DPP status. One interviewee discussed recognition status, saying:

*Coordinator A: Before, the requirements for getting preliminary recognition was simple. It was attendance. For full recognition the weight loss requirements were so strict. So that was another reason why I didn't care to be able to bill Medicaid/Medicare because we are not going to meet [the 5% weight loss] requirement. But now that [Hb]A1C is on the table, it's something to reconsider. Currently we make zero dollars, but maybe in the future, we would.*

Several interviewees discussed having attendees who did not meet eligibility criteria yet allowing them to attend the sessions but not counting them as participants for funding purposes. Such “unofficial” attendees were often family members of someone with pre-diabetes, or community members who were motivated to make lifestyle changes. One program director mentioned how their program handled unofficial attendees, stating:

*Lifestyle Coach B: So, I really didn’t want to decline them to attend the class, because the free class sometimes the funding was provided by New York City Department of Health or as a grant from the CDC. But in that case, we didn't report the undiagnosed or their family members, but we only report [eligible participants to the CDC], but we didn't decline those people.*

##### Opportunities

Theme: Incentivizing participants and operators could increase program reach and success

Healthcare sites, community based organizations, and public hospital sites noted that if billing activities were to be streamlined and centralized, the program may be more likely to be cost effective. Although it was unclear whether any other DPP sites were able to bill for services provided during the pandemic, one interviewee noted that the lifestyle company, was able to continue an established billing structure in which select insurance companies reimbursed participant fees for those who completed a required number of sessions and achieved > 5% weight loss. They noted:

*Director C: We collect maybe half the time and that's worth it for us. So, finances and billable hours is something I always have to be thinking about. We have been fortunate that our organization believes that investing in nutrition education is a worthy endeavor and we bill for all of our services,[including] our nutrition services from insurance companies. Sometimes they reimburse them, sometimes they don't.*

The New York State Department of Health and New York City Department of Health have provided incentives such as water bottles, exercise bands, etc., to reward participants for session attendance and reaching program milestones. However, several interviewees noted that incentivizing participation, goal setting, and lifestyle changes with monetary incentives above and beyond symbolic incentives may increase success rates. One interviewee stated that:

*Coordinator A: If we had more funding and I could be like: if you complete the program, you get a $50 [gift] card or other perks and incentives. I think it could improve show rates and completion rates.*

## Discussion

In this project we sought to document whether and how New York City DPPs adapted and served participants during COVID-19 pandemic lockdowns and identify successes and challenges to operating a DPP remotely. We found that some DPPs were indeed able to persevere despite significant challenges to operating remotely, and that the successes were primarily due to resilient, resolute, and creative staff.

Although we did not interview any DPP participants, the evidence of participant interest, motivation and perseverance was highlighted repeatedly by the DPP staff members. The DPP presents a free opportunity for New Yorkers to prevent the progression to diabetes. However, the program requires significant inputs in the form of staff time, space/ resources, and a high level of self-motivation for participants to succeed.

Despite the DPP’s evidence-based curriculum and demonstrated success in achieving weight loss among some participants, enrollment and retention are both relatively low both in New York City and nationally. Our findings show that the in-person mode of delivery and length of the program may be major obstacles to attendance. Therefore, enrollment and retention may have been the same or even better in DPPs that remained in operation during the pandemic. This presents an opportunity to reexamine the implementation policies. The pandemic demonstrated that to increase participation in the DPP, programs should be flexible and accommodate busy schedules, offer virtual asynchronous or synchronous courses, make cultural adaptations, supplement the workshops with social media communication, provide digital devices and internet access to participants, and offer financial incentives for participants achieving program milestones. A randomized controlled trial demonstrated that compared to a control group, financial individual and group incentives almost doubled attendance rates and increased weight loss achievement by about a third [25].

The lack of sustained funding for the DPP was an unexpected but central topic in our interviews. Although modest startup funds may be available to new DPP host organizations from local and state health departments, the CDC does not provide continued funding for the implementation of the DPP. Host organizations are expected to cover program costs such as staff time, meeting spaces, food, incentives, printing, marketing, etc., and then bill health insurance companies, Medicare/Medicaid, or have participants self-pay. Since 2016, the Centers for Medicare & Medicaid Services (CMS) have certified Medicare DPP as a billable service. Pay-for-performance reimbursement rates are insufficient and can leave an up to $800 deficit per participant between actual cost of implementation and reimbursement [19, 26, 27]. As of December 2019, only 185 or approximately 12% of the more than 1,500 DPP host organizations nationally had enrolled as Medicare DPP providers [28]. Between 2018 and 2021 only 3,600 Medicare beneficiaries had taken advantage of the opportunity. Although evidence suggests that Medicare DPP participants lose weight, the limited duration of the initiative is inadequate to show whether DPP participation lowers healthcare expenditures [13, 29, 30]. In addition to Medicare, eleven states also include the DPP as a covered health benefit for eligible Medicaid participants [28]. Employer sponsored DPPs offer the lifestyle program as an employee benefit, which improves employee health, lowers the risk of diabetes and other chronic disease, while in turn lowering company health insurance expenses [31]. However, our interviews showed that the fee for service may be forfeited because it is not cost effective to seek reimbursements from Medicare, Medicaid, and health insurance companies, or lifestyle coaches and administrators simply do not have the time to submit the documentation necessary for reimbursement. Inadequate funding mechanisms available to sustain DPPs make them vulnerable to cost–benefit-based business decisions [32]. There is an urgent need for more robust funding mechanisms to support the DPP, a challenge which is recognized by the host organizations as well as the city and state departments of health, and the CDC.

In addition, in our study several organizations demonstrated that their DPPs can be accessible both virtually and in person. This opens a unique opportunity to participants with shared cultural backgrounds who are not living in the same geographic location.

### Strengths and limitations

This study has several strengths. It is one of few studies representing the perspectives of DPP lifestyle coaches and program management rather than participant outcomes. Specifically, this study provides unique firsthand perspectives on participant recruitment, retention, workshop implementation, data collection, and funding during a global public health emergency.

The study has two major limitations. Of the 47 sites in New York City, we only succeeded in reaching staff from 8 organizations and interview representatives from 7 of those. In comparison, the CDC conducted a similar study in which they interviewed staff from 5 DPP affiliates nationally to assess the challenges of adjusting to the COVID-19 public health emergency [16]. Nevertheless, it is unknown how many of the 47 sites were able to focus on the DPP during the COVID-19 lockdowns. Most New York City DPP host organizations are hospitals, clinics, medical centers, or community-based organizations which quickly had to reassign staff, space, and resources to address urgent COVID-19 needs (e.g., testing, contact tracing, hospitalization, food distribution, childcare, homeschooling, bereavement, housing insecurity). In fact, the CDC recognizes that as of spring 2023 many DPP host organizations had yet to resume activities after COVID-19 [15]. Secondly, we interviewed staff from only one program which failed to deliver DPP during the COVID-19 pandemic. Unfortunately, we were unable to interview DPP organizations which did not continue. Those organizations may have been able to provide additional insights about why and how programs failed to sustain the DPP. Therefore, these findings primarily represent programs that persevered and successfully delivered DPP program activities during the COVID-19 pandemic. We focused our study on New York City DPPs and therefore these findings represent this setting only. Yet, the sites represent a diverse set of host organizations and experiences and many of our findings confirm those of other local or national studies examining similar research questions [16–18].

## Conclusions

The NYC DPPs we interviewed suffered greatly during COVID-19 lockdowns and limits on social gathering because program staff and participants were unprepared for virtual classes. Nevertheless, most were successful due to resilient, dedicated, and extraordinarily creative staff. The study findings will aid the CDC, New York City Department of Health, New York State Department of Health, funders, and host organizations to inform ways to optimize DPP programs in virtual settings as well as options for conducting health education programs during public health emergencies, natural disasters, and other disruptive or geographically isolating circumstances. The pandemic highlighted opportunities for successful virtual DPPs in rural and urban settings, and reiterated the need for more robust funding mechanisms, staff support, and technical assistance to ensure sustainability and scalability of the DPP.

### Supplementary Information

**Additional file 1.**

**Additional file 2.**

**Additional file 3.**

## Data Availability

The datasets generated and/or analyzed during the current study are not publicly available. To protect the privacy and confidentiality of study participants, quotes from interviews are deidentified. However, providing access to full transcripts could increase the risk of deductive identification of participants. Therefore, the data cannot be made publicly available. Data may be made available upon special request by contacting Margrethe F. Horlyck-Romanovsky, DrPH, at margrethehr@brooklyn.cuny.edu.
