# Supplementary material for: Staff resilience and innovation essential to New York City diabetes prevention programs going virtual during COVID-19 pandemic lockdowns
Source: BMC Health Serv Res. 2023 Oct 25;23:1148. doi: 10.1186/s12913-023-10129-y (PMC10599031; doi:10.1186/s12913-023-10129-y)
Supplement: Supplementary file 1 — Additional file 1. [file 12913_2023_10129_MOESM1_ESM.pdf]

Supplementary File 1

Diabetes Prevention Programs in NYC study: Intake Form

CUNY Universal Integrated IRB Protocol: 2021-0194, Approved: 3/12/2021
Evaluation of Diabetes Prevention Programs in NYC during COVID-19

* Required

About you!

1. Please enter your six digit code (to protect your privacy we will only collect your assigned code). *

____

2. Indicate your role at this DPP. *

Mark only one oval.

- ( ) Director
- ( ) Coordinator
- ( ) Lifestyle Coach
- ( ) Other: ____

3. How long have you been a director, coordinator, lifestyle coach, and/or other persons involved in the recruitment, implementation or leadership of DPP(s)? *

____

4. Please list any credentials you may have. (eg. RD/RDN, CHES, CDCES, RN)

____
____
____
____
____

DPP Operations

5. What type of organization(s) hosts your DPP? Check all that apply. *

Check all that apply.

- [ ] Hospital
- [ ] Out-patient Clinic
- [ ] Community-Based Organization (CBO)/Non-profit
- [ ] Medical Practice
- [ ] Private/For-profit company (eg. Weight Watchers, Noom)
- [ ] Religious
- [ ] Other: ____

6. What class type(s) do you offer? Check all that apply. *

Check all that apply.

- [ ] Public
- [ ] Employee
- [ ] Member-only
- [ ] Other: ______________________

7. As a result of the pandemic, were sessions suspended at any point? *

Mark only one oval.

- ( ) Yes
- ( ) No

8. If yes, how long were sessions suspended?

____

9. Before the pandemic, what was your DPP session delivery mode? *

Check all that apply.

- [ ] In-Person (delivered 100% in-person for all participants by trained Lifestyle Coaches)
- [ ] Online (delivered 100% online for all participants; independent learning)
- [ ] Distance Learning (delivered 100% by trained Lifestyle Coaches via remote classroom or telehealth (i.e., conference call or Skype))
- [ ] Combination (delivered as a combination of any of the previously defined delivery modes (1. – 3. above))

10. Did the delivery mode change as a result of the ongoing pandemic? *

Mark only one oval.

- ( ) Yes
- ( ) No *Skip to question 12*

DPP Operation Changes

11. What delivery mode did you adopt as a result of the ongoing pandemic? Check all that apply. *

Check all that apply.

- [ ] In-Person (delivered 100% in-person for all participants by trained Lifestyle Coaches)
- [ ] Online (delivered 100% online for all participants; independent learning)
- [ ] Distance Learning (delivered 100% by trained Lifestyle Coaches via remote classroom or telehealth (i.e., conference call or Skype))
- [ ] Combination (delivered as a combination of any of the previously defined delivery modes (1. – 3. above))
- [ ] Other: ______________________

DPP Participants

12. Before the pandemic, how many DPP participants did you typically serve each year? (Provide a single whole number, eg. 25) *

____

13. Before the pandemic, how did participants hear about your program? Check all that apply. *

Check all that apply.

- [ ] Advertisements
- [ ] Community based organization referrals
- [ ] Federally Qualified Health Centers referrals
- [ ] Hospital referrals
- [ ] In-person outreach/flyers
- [ ] Medical office (private) referrals
- [ ] Social media
- [ ] Word of mouth
- [ ] Other: ______________________

14. As a result of the ongoing pandemic, how do participants currently hear about your program? Check all that apply. *

Check all that apply.

- [ ] Advertisements
- [ ] Community-based organization referrals
- [ ] Federally Qualified Health Center referrals
- [ ] Hospital referrals
- [ ] In-person outreach/flyers
- [ ] Medical office (private) referral
- [ ] Social media
- [ ] Word of mouth
- [ ] No changes to outreach methods
- [ ] Other: ______________________

15. As a result of the ongoing pandemic, DPP participation has... *

Mark only one oval.

- ( ) increased
- ( ) decreased
- ( ) stayed the same

16. List the main racial/ethnic/cultural groups that attend your DPP. Be as specific as possible. (eg. Black American, Cambodian, Chinese, Chilean, French, Garifuna, Ghanaian, Honduran, Jamaican, Nigerian, Puerto Rican, Russian, White American) *

____

____

____

____

____

17. On average, what percentage of your DPP participants are foreign-born? (Provide a whole number, eg. 50%) *

____

This content is neither created nor endorsed by Google.

Google Forms
