# Supplementary material for: Staff resilience and innovation essential to New York City diabetes prevention programs going virtual during COVID-19 pandemic lockdowns
Source: BMC Health Serv Res. 2023 Oct 25;23:1148. doi: 10.1186/s12913-023-10129-y (PMC10599031; doi:10.1186/s12913-023-10129-y)
Supplement: Supplementary file 3 — Additional file 3. [file 12913_2023_10129_MOESM3_ESM.docx]

**Supplementary Table 1. Diabetes Prevention Program Interview Quotes, Themes Organized by Domain**

| **Category** | **Challenges** | **Opportunities** |
| --- | --- | --- |
| **Going Virtual** | Coordinator A*: I will not run a program unless I’m running it in person, because it just was not successful with our population and the time we have. [...] If you want to do it virtually you need time beforehand to practice with each and every person to make sure they can log on. [...] I want it to be in person, I like the community feel that it promotes. It’s not supposed to be a lecture, it’s supposed to be a roundtable discussion.*  Lifestyle Coach B*: In in-person class, they are very active [they talk about themselves and how they are really concerned about their diabetes] or their successes [...] but in virtual class they just listen. It’s a little hard [because they still have difficulty operating Zoom and the mute function.]*  Lifestyle Coach A*: It’s the little things that [would keep people interested] and that we had to omit because of COVID. Given that the program would have people more engaged, [we would] take a trip to the supermarket and or farmers market [or] bring healthy snacks in for them.* | Lifestyle Coach B*: We have Brooklyn clients, we have Staten Island clients [they’re able to access the DPP] remotely because it is a virtual classroom.*  Lifestyle Coach D*: A lot of these participants [are somewhat tight with money and even commuting to one of the centers could be a bit of a challenge… The flexibility [of online classes allows them] to take the class during their lunch hour or while preparing meals.*  Director C*: We came into the pandemic [and] we found out that there was the option to do [the DPP] virtually. We had started doing some virtual classes already for our patients, [like] virtual yoga for free, virtual cooking classes, and we had success with attendance. [...] We were surprised that we got a good cohort, and not only that, but they were consistently coming.*  Coordinator B*: Especially for younger populations like it's, In full disclosure, I will say I'm a millennial  in my mid-30s. I can’t imagine going to a conference room at a hospital or Y facility for an hour and a half class for a year.* |
| **Staff** | Lifestyle Coach E*: I am a dietitian and I have a full clinic to cover. So the timing is tough, [...] I only spend once a month to call the participants. The other lifestyle coach is a nurse assistant [...] he was at a COVID floor so he couldn’t do any of this with me.*  Coordinator A*: Not being able to control [my] staff’s schedules, like you can’t run a program for an hour and think that’s the only time you need for the program. You need prep, you need follow up. You need to have time and [...] and they’re just not given admin time [and] it’s awful.*  Lifestyle Coach B*: Only two coaches are not enough. [...] Even before the pandemic we needed to work with four coach members.*  Coordinator A*: Say we had like 15 people. Someone needs to call 15 people and say, have you used Web X or whatever platform, [...] are you comfortable with it. [...]because it takes so much time. [...] I think clerical type support would be the best.*  Lifestyle Coach A*: We have four people who are trained lifestyle coaches. We were supposed to get a few more trained this year, but because of the pandemic it just didn’t happen. We are aiming to get more people trained hopefully by the beginning of next year.* | Lifestyle Coach B*: It was a great teamwork and then each lifestyle coach was passionate even though it's very hard…New York city [was] hardest hit by the Covid-19, but even though we really a little bit scared to go out to meet someone in person [...] we were brave and courageous.* |
| **Training** | Lifestyle Coach B*: In late March, we had a virtual coach meeting so we decided to take responsibility [on]how to work with the clients. But even though the coach members around the 60 years late 50 and 60 so this why they could understand how difficult they even though they didn't know what Zoom is. As we start to get into character to familiar with the Zoom, Zoom hosting, Zoom operation. Maybe something like “The lifestyle coaches were around 60 years old, late 50s, so it was difficult to train them. They didn’t know what Zoom was, and it took a while to get familiar with Zoom. - I think this was a manual edit/comment that we didn’t change?* | Lifestyle Coach A*: The Department of Health and Mental Hygiene [have] helped us tremendously. They got us trained and I actually have [monthly meetings with] one of their workers. She helped me with the process of getting certified for Medicaid. Any time I have any issues, whenever I have to submit data to the CDC they’re great so, they got us trained and they’re still here, helping us.*  Lifestyle Coach B*: To do this we need to get training from one of our younger staff who are already familiar with Zoom so we coaches receive the training.* |
| **Recruitment, Retention, and Communication** | Lifestyle Coach A*: [People] couldn't get an appointment with their doctor, so they didn't remember when their last A1C was. That was a little more challenging and that's why we didn't get as many participants because we didn’t have a lot of referrals.*  Lifestyle Coach C*: We started with about 15 patients, it’s down to 8 now [...] I think that they’re really enjoying it, they’re coming to most of the sessions. For our clientele, there’s a bunch of different things going on in their lives, so it’s really hard for them to commit long term.*  Lifestyle Coach E*: We started with phone calls. I even mailed them the curriculum, the modules, I mailed the modules and I even mailed them the health bucks because farmers markets [were] still on.*  Coordinator A*: I want to make sure I’m signing up with your email address, and I want to know, do you use your email. Like, I want to know that I can find you outside of here. That’s just a step in the right direction towards if we needed to go virtual again, like [if I know your email] that tells me that you probably can do a Web X or other means of communication. The [certified lifestyle coach] CLC last time, she was putting stuff in the mail all the time and it’s so laborious compared to just sending an email with attachments.*  Director B*: The other issue that comes up is that somebody sent me something on my phone, [someone goes through the Google telephone number, [someone sends] me stuff on my cell number, then I have some people who will email [me].*  Lifestyle Coach E*: It’s very tough because now we don’t have the [white] board there’s nothing visual. Most of our participants are seniors, [so if] you talk too long on the phone you don’t know if they are understanding, or if they are really listening. [...] So I actually mailed them all the modules ahead of time and asked them, did you receive it and read it?* | Director C*: We were surprised that we got a good cohort and not only that, but they consistently are coming, and so it did show a huge difference compared to anything that we've ever tried to do in person at the clinics - so far it has been a … fortunate event.*  Coordinator C*: [When a physician says] “Oh, I heard you’re in the DPP and you’re doing so well” - I feel like that has really helped with retention when the doctor is involved in the care. Other than that, like ethnic groups, it really depends. Age, too, is kind of all over the place. It really depends on the motivation of the person. Sometimes, too, how the class gels [is important]. Some people in some classes become friends and they’re like “[name of participant], you weren’t here last week, what happened to you?”*  Lifestyle Coach D*: participation is a huge factor [to succeed] in the program. Sometimes [I] get one or two that are super shy, so I make sure to give them my direct line. [I say] Please give me a call and I will work with you every step of the way, so I make myself available as much as I can to make sure that they succeed in the program. [...] I feel very passionate about this program, and I give it my all.*  Director A*: We are on a social media [app], you know, like all [our participants] have something called [name of app], I don't know if you heard of it.[...] everybody is on [the app]. So, you know, like in DPP you know we have like a group of chatting room, so they were all in chatting room already, so we just have to tell let them know we got to start. You know where we start the class.*  Director A*: Yeah, everybody was so excited, and they were like try to make the healthy plate and take the picture and show it. Oh, I made this and the coaches [would] make a comment “wow that looks good!”, “that’s beautiful!”* |
| **Educational Materials and Workshops** | Lifestyle Coach A*: It’s the little things that [would keep people interested] and that we had to omit because of COVID. Given that the program would have people more engaged, [we would] take a trip to the supermarket and or farmers market [or] bring healthy snacks in for them.*  Coordinator B*: All of our materials are in English, some [are in] Spanish. That is a barrier having, more specific kinds of languages we don’t necessarily have materials available, [like] in Mandarin or Vietnamese.* | Coordinator B*: “There’s kind of a hole in our materials in terms of language. There are lots of articles and cheat sheets, where there’s a lot of resources available [to adapt] to more culturally specific means. So, highlighting for members saying “Hey, if you’re looking for a healthier spin on empanadas, here are these recipes in the database, and you can look for [a] specific cuisine. But there are plenty of participants who are looking for recipes or information [within] the [name of company] system. There are lots of articles and cheat sheets, where there’s a lot of resources available [to adapt] to more culturally specific means. [We] even [did] more like this past month, looking at Hispanic Heritage Month, and like that’s been a focus of a lot of the virtual and in-person workshops. So, looking at that, and really highlighting for members saying “Hey, if you’re looking for a healthier spin on empanadas, here are these recipes in the database, and you can look by [a] specific cuisine.”*  Coordinator A*: They’re going to put their own spin on things, like you’re supposed to bring your own personality [and] perspective and cultural sensitivity to these programs. The people that were teaching the Spanish class are Spanish, so they know the foods [to keep in] conversation.*  Lifestyle Coach D*: Definitely healthy eating or showing them how to prepare healthy meals and with healthy meals, showing them how to prepare [something] basic like a healthy shake. I feel like individuals don’t know how serious diabetes can be, so perhaps bringing a professional, such as a primary care doctor, [to] join in on some of these sessions and [speak] about the seriousness of diabetes. I don’t want to say scare the participants, but something that wakes them up.* |
| **Data Collection/ Tracking of Weight** | Director B*: That has been ironically, one of the most challenging parts of this. Getting people used to sending in your weights and activities minutes. It’s a nightmare, and I’ve even invested in the apps which is one of the programs where [I] personally put all the information in right after the session.*  Coordinator B*: The challenge is making sure we have all that data [...]. Self-reporting weight [is] our proxy for attendance. The caveat to that was obviously folks who don’t have a scale at home. [...] There was an accuracy issue as well, so if you miss one week that throws all of your data off. Then we get a flag from the CDC saying you know this person says they lost and gained 75 pounds, [and] that throws off your 5%.”*  Coordinator A*: I understood why they did it because of all the Community based organizations, not being able to always have A1C’s. But to not have it as an option, I thought was such a disservice because we had such great statistics of like literally curing their pre diabetes and it didn't matter because they only lost two pounds”* | Coordinator B*: There are several health insurance companies that will not accept self-reported weight but they would accept Bluetooth scales sync to an app.*  Coordinator C*: [affiliated clinics] and they would help do free A1C tests, which was really helpful especially [since] we did them week 1 and week 16. At the very end, so people can kind of see the progress, and it was helpful with the doctors.* |
| **Funding** | Coordinator A*: Before, the requirements for getting preliminary recognition was simple. It was attendance. For full recognition the weight loss requirements were so strict. So that was another reason why I didn't care to be able to bill Medicaid/Medicare because we are not going to meet [the 5% weight loss] requirement. But now that [reduction in Hb]A1C is on the table, it's something to reconsider. Currently we make zero dollars, but maybe in the future, we would.*  Lifestyle Coach B*: So, I really didn’t want to decline them to attend the class, because the free class sometimes the funding was provided by New York City DOH or as a grant from the CDC. But in that case, we didn't report the undiagnosed or their family members, but we only report [eligible participants to the NYSDOH and the CDC]but we didn't decline those people.* | Director C*: We collect maybe half the time and that's worth it for us. So, finances and billable hours is something I always have to be thinking about. We have been fortunate that our organization believes that investing in nutrition education is a worthy endeavor and we bill for all of our services,[including] all of our nutrition services from insurance companies. Sometimes they reimburse them, sometimes they don't.*  Coordinator A*: If we had more funding and I could be like: if you complete the program, you get a $50 [gift] card or other perks and incentives. I think it could improve show rates and completion rates.* |
